# From SGAP-Model to SGAP-Score: A Simplified Predictive Tool for Post-Surgical Recurrence of Pheochromocytoma

**DOI:** 10.3390/biomedicines10061310

**Published:** 2022-06-03

**Authors:** Mirko Parasiliti-Caprino, Fabio Bioletto, Chiara Lopez, Martina Bollati, Francesca Maletta, Marina Caputo, Valentina Gasco, Antonio La Grotta, Paolo Limone, Giorgio Borretta, Marco Volante, Mauro Papotti, Anna Pia, Massimo Terzolo, Mario Morino, Barbara Pasini, Franco Veglio, Ezio Ghigo, Emanuela Arvat, Mauro Maccario

**Affiliations:** 1 Endocrinology, Diabetes and Metabolism, Department of Medical Sciences, University of Turin, 10126 Turin, Italy; mirko.parasiliticaprino@unito.it (M.P.-C.); chiara.lopez@unito.it (C.L.); martina.bollati@unito.it (M.B.); valentina.gasco@unito.it (V.G.); ezio.ghigo@unito.it (E.G.); mauro.maccario@unito.it (M.M.); 2 Department of Oncology, University of Turin, 10043 Orbassano, Italy; francesca.maletta@unito.it (F.M.); marco.volante@unito.it (M.V.); mauro.papotti@unito.it (M.P.); 3 Pathology Unit, City of Health and Science University Hospital of Turin, 10126 Turin, Italy; 4 Department of Health Sciences, University of Eastern Piedmont, 28100 Novara, Italy; marina.caputo@med.uniupo.it; 5 Endocrinology and Hypertension, Cardinal Massaia Hospital, 14100 Asti, Italy; lagrotta@asl19.asti.it; 6 Endocrinology, Diabetes and Metabolism, A.O. Ordine Mauriziano, 10128 Turin, Italy; plimone@mauriziano.it; 7 Endocrinology and Metabolism, Santa Croce and Carle Hospital, 12100 Cuneo, Italy; giorgio.borretta@unito.it; 8 Pathology Unit, San Luigi Gonzaga University Hospital, 10043 Orbassano, Italy; 9 Internal Medicine, Department of Biological and Clinical Sciences, University of Turin, 10043 Orbassano, Italy; a.pia@sanluigi.piemonte.it (A.P.); massimo.terzolo@unito.it (M.T.); 10 Surgery, Department of Surgical Sciences, University of Turin, 10126 Turin, Italy; mario.morino@unito.it; 11 Medical Genetics, Department of Medical Sciences, University of Turin, 10126 Turin, Italy; barbara.pasini@unito.it; 12 Internal Medicine and Hypertension Unit, Department of Medical Sciences, University of Turin, 10126 Turin, Italy; franco.veglio@unito.it; 13 Oncological Endocrinology, Department of Medical Sciences, University of Turin, 10126 Turin, Italy; emanuela.arvat@unito.it

**Keywords:** pheochromocytoma, chromaffin system, predictive score, recurrence prediction, machine learning

## Abstract

A reliable prediction of the recurrence risk of pheochromocytoma after radical surgery would be a key element for the tailoring/personalization of post-surgical follow-up. Recently, our group developed a multivariable continuous model that quantifies this risk based on genetic, histopathological, and clinical data. The aim of the present study was to simplify this tool to a discrete score for easier clinical use. Data from our previous study were retrieved, which encompassed 177 radically operated pheochromocytoma patients; supervised regression and machine-learning techniques were used for score development. After Cox regression, the variables independently associated with recurrence were tumor size, positive genetic testing, age, and PASS. In order to derive a simpler scoring system, continuous variables were dichotomized, using > 50 mm for tumor size, ≤ 35 years for age, and ≥ 3 for PASS as cut-points. A novel prognostic score was created on an 8-point scale by assigning 1 point for tumor size > 50 mm, 3 points for positive genetic testing, 1 point for age ≤ 35 years, and 3 points for PASS ≥ 3; its predictive performance, as assessed using Somers’ D, was equal to 0.577 and was significantly higher than the performance of any of the four dichotomized predictors alone. In conclusion, this simple scoring system may be of value as an easy-to-use tool to stratify recurrence risk and tailor post-surgical follow-up in radically operated pheochromocytoma patients.

## 1. Introduction

Pheochromocytomas are rare neuroendocrine tumors that arise from adrenomedullary chromaffin cells. Commonly, they produce one or more catecholamines, but they may be biochemically silent in some cases [1,2]. It is important to correctly diagnose and treat these tumors for several reasons since they are associated with significant complications and adverse outcomes [3,4,5]: if untreated, unopposed catecholamine hypersecretion remarkably increases the cardiovascular morbidity and mortality; moreover, pheochromocytomas may enlarge with time and may cause mass-effect symptoms, with possible extension into adjacent tissues; some of them may also have malignant potential, defined as the possible development of distant metastases in nonchromaffin tissues; and finally, a correct diagnosis and case detection may help to identify and address syndromic/familial forms [1,2,3,4,5,6].

The treatment of choice is represented by surgery, which can be curative in most cases [1,2]. However, even after radical resection of the tumor, the risk of recurrence is still not negligible [1,2,3,7,8], with an estimated recurrence rate of 1% every year [3]. In patients with a completely resected pheochromocytoma, the definition of disease recurrence comprises three main categories [2,3]: (i) a local relapse, defined as the recurrence of disease in the primary tumor site; (ii) distant metastases, defined as the recurrence of disease in distant non-chromaffin tissues; and (iii) a new primary tumor, defined as the re-occurrence of disease in other chromaffin-derived tissues, such as the contralateral adrenal gland.

Given the clinical relevance of the disease, a reliable prediction of the recurrence risk would be of key importance in tailoring patients’ follow-up. In recent years, several factors have been found to play a prognostic role regarding this outcome. First of all, pheochromocytoma recurrence risk was shown to be associated with some simple clinical features, such as younger age [7,9,10,11], larger tumor size [7,9,12,13,14,15,16], and noradrenergic secretory phenotype [7,9,11,17]. Another important prognostic factor for the prediction of recurrence risk is the genetic status. Currently, a genetic germline cause can be identified in around 40% of pheochromocytomas [2,18]. Patients with familial forms were widely demonstrated to be at higher risk of disease recurrence compared with those with sporadic neoplasms [2,3,6,7,12,18,19,20]; in particular, germline mutations in known pheochromocytoma-related susceptibility genes are remarkably associated with an increased risk of local recurrence or metachronous tumors. Nevertheless, depending on the mutated gene, they may be linked with an increased malignant potential too [4,21,22]. Histopathological features were also examined as potential predictors for recurrence and, in particular, for metastatic disease. In the last two decades, three scoring systems were developed to identify tumors with malignant biological behavior: the Pheochromocytoma of the Adrenal gland Scaled Score (PASS) [23], the Grading system for Adrenal Pheochromocytoma and Paraganglioma (GAPP) [24] and its subsequently modified version (M-GAPP) [25], and the COmposite Pheochromocytoma/paraganglioma Prognostic Score (COPPS) [26]. Notably, differently from PASS, these latter scoring systems (GAPP, M-GAPP, and COPPS) do not only include classical histopathological parameters but also comprise biochemical data about hormonal secretion [24,25] and immunohistochemical results for SDHB [25,26] and PS100 [26] staining; this acknowledges the necessity of further non-histological parameters to reach a better predictive performance.

Overall, even if several studies explored the association of all these predictors with recurrence, reliable tools for an integrated multivariable estimation of pheochromocytoma recurrence risk after radical surgery are still scarce. Recently, our research group developed and internally validated a multivariable model for this purpose [27], which was named SGAP-model (Size, Genetic, Age, and PASS) due to the predictive factors that were finally included. To the best of our knowledge, this was the first prognostic tool that integrated all the most relevant features (i.e., genetic, histopathological, and clinical patient data) for the post-surgical prediction of pheochromocytoma recurrence risk. However, though showing a good predictive accuracy, its use in clinical practice might be hampered by the complex formula underlying the estimation of the outcome, as it handles the included variables as continuous and requires a calculator for the final risk estimation. In light of this, the aim of the present study was to simplify this tool to a discrete score in order to allow for easier clinical use.

## 2. Materials and Methods

### 2.1. Design

The present study enrolled all eligible patients diagnosed with pheochromocytoma between 1990 and 2016 in nine centers with recognized expertise in adrenal gland disorders in Piedmont, a region in Northwest Italy. Pertinent data were collected from prospective registries and analyzed retrospectively. The eligibility criteria were: (i) confirmed diagnosis of pheochromocytoma; (ii) radical surgery with an apparent cure, defined as R0 resection confirmed at a pathological examination, absence of other disease localizations, and hormonal normalization at 6 weeks after surgery; (iii) availability of histological specimen for PASS evaluation; and (iv) appropriate post-surgical follow-up on at least an annual basis.

Approval from local ethics committees was obtained for the analysis of patient data in all centers with central coordination by the Ethics Committee of the City of Health and Science University Hospital of Turin. Written informed consent was obtained from all patients.

### 2.2. Study Population and Data Collection

For each patient, the following information was collected: (i) personal and clinical data (gender, age at diagnosis, familial history of pheochromocytoma or pheochromocytoma-associated syndromic forms); (ii) biochemical data (plasma and/or 24 h urinary catecholamines/metanephrines, plasma chromogranin A); (iii) imaging data (tumor size and side at conventional MRI or CT imaging, radiotracer uptake at functional imaging); (iv) genetic testing and mutation type; (v) date of surgery; and (vi) histopathological features.

Histopathological specimens were reviewed by a single expert pathologist at the time of study completion (F.M.), who was always assisted by at least another early career colleague with a specific focus on PASS re-evaluation. Genetic testing was performed on blood samples based on clinical indication (i.e., positive familial history and/or other specific clinical or histological risk features). In fact, the indication to perform genetic testing in all patients on a routine basis was introduced only in recent years, after the publication of the Endocrine Society 2014 guidelines [1] and European Society of Endocrinology 2016 guidelines [2]. Samples collected for genetic testing were periodically re-evaluated to search for new mutations discovered in more recent years, as well as to possibly reclassify variants of uncertain significance (VUS) that were shown to be pathogenic in time. The following genes were evaluated for possible germline mutations: RET, VHL, NF-1, SDH-AF2, SDH-A, SDH-B, SDH-C, SDH-D, TMEM-127, MAX, EPAS-1, and FH.

All patients were followed-up annually using hormonal and/or imaging assessments as appropriate according to the secreting status of the original tumor. Recurrence was defined as a local relapse, new primary tumor, or distant metastases, as detected using CT/MRI and/or functional imaging. The date of last follow-up visit, development and type of recurrence, disease-free survival time, and cause and date of death were collected for all patients.

### 2.3. Statistical Analysis

Baseline characteristics of all patients included in the analysis were summarized using the mean and standard deviation for continuous variables and percent values for categorical data. The follow-up duration was summarized using the median and interquartile (IQR) range.

In our previous paper [27], predictors were assessed for inclusion in a multivariable Cox regression model using a backward selection algorithm; the retrieved model was then internally validated on 1000 bootstrap samples. In the present study, in order to facilitate the use of the model in clinical practice, a simplified predictive tool was derived through the dichotomization of continuous predictors. The choice of the cut-points to be used was based on existing data in the literature whenever possible, or on a combination of clinical reasoning and a supervised machine-learning approach in the other cases. Multivariable Cox regression was re-applied on discretized variables for the creation of this simplified model. A discrete-point score was retrieved by assigning integer point scores to each predictor upon normalization and rounding of regression β-coefficients. The predictive performance of the score was evaluated using Somers’ D and compared to those of its single components alone [28]. As a final step, a classification and regression tree (CART) algorithm was applied to group the risk classes of clinical relevance.

Results were considered statistically significant if *p* < 0.05. Statistical analysis was performed using R 4.1.1 (R Foundation for Statistical Computing, Vienna, Austria). For the supervised machine-learning approach used for score creation, the “discretization”, “survival”, “survMisc”, “rpart”, and “rms” packages were used. Kaplan–Meier curves were generated using STATA 17 (StataCorp, College Station, TX, USA).

## 3. Results

### 3.1. Characteristics of the Study Population and Brief Description of the SGAP-Model

As already described in our previous study, a total of 177 patients fulfilled the inclusion criteria. Overall, 89 (50.3%) of them were male and 88 (49.7%) were females. The mean age at diagnosis was 49.6 ± 16.4 years. The median follow-up was equal to 79 (IQR 50-119) months. Full details about the study population are presented in the reference article [27].

As previously reported [27], after Cox regression and the application of a backward selection algorithm, the variables selected as independent predictors of recurrence were tumor size, positive genetic testing, age, and PASS. Due to the included parameters, this model was named SGAP-model (Size, Genetic, Age, and PASS). The final model equation estimating the overall recurrence risk could be computed as S_0_(t)^exp(z)^, where z = 0.0124 × tumor size (in mm) + 1.6367 × genetic status (1 if positive, 0 otherwise) − 0.0309 × age (in years) + 0.1456 × PASS; the value of S_0_(t) represents the baseline survival at time t (i.e., the estimated recurrence-free survival if all predicting variables were hypothetically set to 0), and was equal to S_0_(2) = 0.9654 at 2 years, S_0_(5) = 0.9382 at 5 years, and S_0_(10) = 0.8807 at 10 years. The predictive performance of this model, as evaluated using Somers’ D, was equal to 0.594 and was significantly higher than the Somers’ D value of any single predictor alone (*p* = 0.002 compared with tumor size; *p* = 0.004 compared with genetic status; *p* = 0.048 compared with age; *p* = 0.006 compared with PASS). Internal validation using bootstrapping techniques estimated an optimistic bias of 6.3%, which was reassuring about a small tendency toward overfitting.

**Figure 1:**
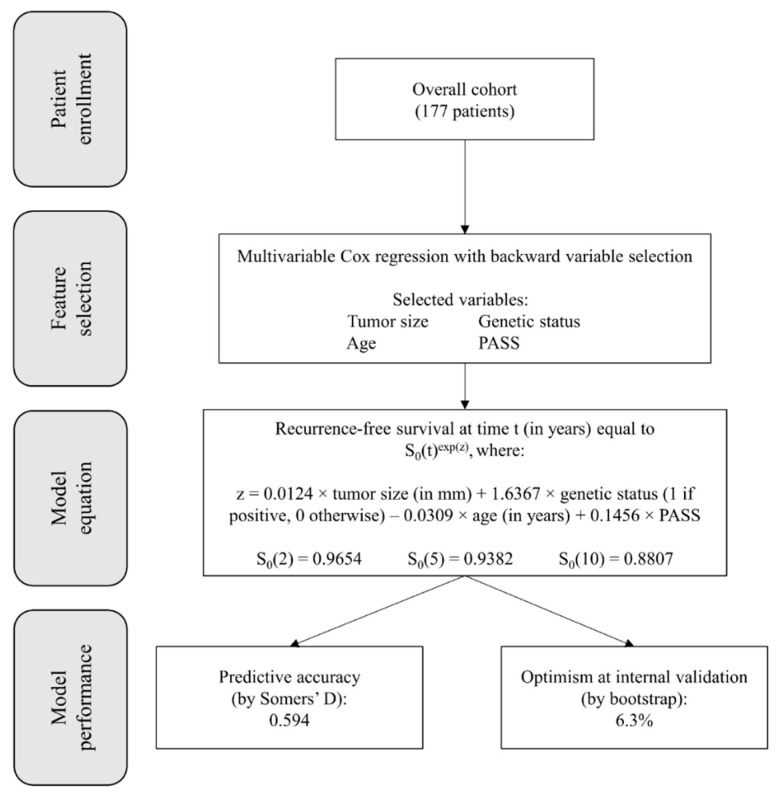
Graphical summary of the process adopted for the development and internal validation of SGAP-model in our previous article. Abbreviations: PASS, Pheochromocytoma of the Adrenal gland Scaled Score; SGAP, Size–Genetic–Age–PASS.

The whole process of development and internal validation of the SGAP-model in our previous article is graphically summarized in Figure 1.

### 3.2. Development of the SGAP-Score

In order to facilitate the use of the model in clinical practice, we proposed a simplified predictive tool that was obtained through the dichotomization of continuous predictors. For the age at diagnosis, we adopted a cut-off of ≤35 years, as already proposed by Cho et al. [9] and by Kim et al. [10]. With respect to tumor size, the frequently proposed cut-off of >50 mm was chosen [11,12,29,30]. For histopathological scoring according to PASS, while the well-accepted cut-off of ≥4 was extensively adopted for the prediction of malignant behavior, no data are actually available about a cut-point to be used for the prediction of recurrence of any type. Using clinical reasoning, it may be argued that a slightly lower cut-off would be a better-performing choice for this scope; this was confirmed by our data in which the cut-off of ≥3 was the one maximizing separation between the classes according to dichotomization algorithms from the survMisc R-package, and was thus chosen for subsequent analyses.

After the dichotomization of the variables, a multivariable Cox regression was re-applied to the discretized variables for the derivation of a simplified predictive model. The retrieved equation for the estimation of recurrence-free survival was equal to S_0_(t)^exp(z)^, where z = 0.4874 × tumor size category (1 if >50 mm, 0 otherwise) + 1.6617 × genetic status (1 if positive, 0 otherwise) + 0.5838 × age category (1 if ≤35 years, 0 otherwise) + 1.4945 × PASS category (1 if ≥3, 0 otherwise); S_0_(t) was equal to S_0_(2) = 0.9927 at 2 years, S_0_(5) = 0.9868 at 5 years, and S_0_(10) = 0.9751 at 10 years. Integer point scores were thus assigned to each predictor upon normalization and rounding of regression β-coefficients, as reported in Table 1. The retrieved score was named SGAP-score and was defined by the sum of all four components as an 8-point scale. Its predictive performance, as assessed using Somers’ D, was equal to 0.577 and was higher than the performance of any of the four dichotomized predictors alone (*p* < 0.001 compared with tumor size > 50 mm; *p* = 0.033 compared with positive genetic testing; *p* = 0.004 compared with age ≤ 35 years; *p* < 0.001 compared with PASS ≥ 3) (Table 2). This corresponded to a concordance index (i.e., Harrell’s C-index) of 0.789, meaning that for any possible pair of patients, the one with the lower SGAP-score had a longer recurrence-free time in 78.9% of cases.

**Figure 2:**
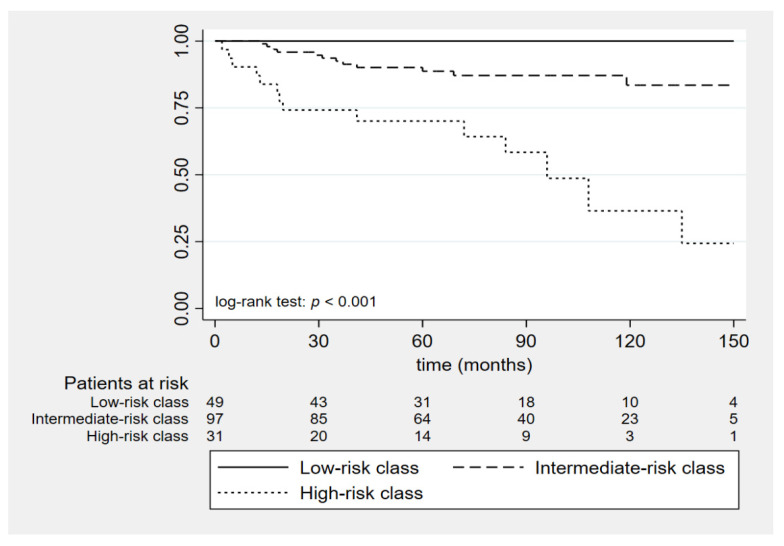
Kaplan–Meier curves for recurrence-free survival according to SGAP-score risk classes. Abbreviations: SGAP, Size–Genetic–Age–PASS.

As a final step, in order to simplify the interpretation of SGAP-score and to identify the most clinically relevant risk classes, a classification and regression tree (CART) algorithmic approach was used. In order to avoid overfitting, a dimensional constraint on terminal leaves was used (>10 patients per terminal leaf). CART algorithm was able to cluster the observations in three clinically relevant risk classes (0–2 points, 3–4 points, 5–8 points). Given their clinical correlates, we referred to these classes as “low-risk”, “intermediate-risk” and “high-risk”. As reported in Table 3, the low-risk class comprised 49 patients and apparently showed no risk of recurrence, even in the long-term (100% of recurrence-free survival both at 5 years and at 10 years). The intermediate-risk class comprised 97 patients and showed a recurrence-free survival of 89% at 5 years and 84% at 10 years. The high-risk class comprised 31 patients and showed a recurrence-free survival of 70% at 5 years and 37% at 10 years. This clear-cut distinction between the recurrence risks could also be seen using the Kaplan–Meier curves, as reported in Figure 2 (*p*-value < 0.001 using the log-rank test).

## 4. Discussion

In the present study, we proposed a practical scoring system for the prediction of post-surgical recurrence risk in radically resected pheochromocytoma based on the integration of genetic, histopathological, and clinical data. A reliable prediction of the recurrence risk of pheochromocytoma after radical surgery would be a key element for the tailoring of post-surgical follow-up. Regarding this scope, our research group recently proposed a multivariable prediction model, which was named SGAP-model due to the included variables (Size, Genetic, Age, and PASS). This model was developed and internally validated, and its predictive performance was significantly higher than those of any single predictor alone. Nevertheless, an obstacle toward the routine employment of the SGAP-model was certainly represented by the complex formula underlying the estimation of the outcomes; though an online calculator was provided for this task, this issue might still represent a relevant limit for the comfortable use of this tool.

In light of this, in the present study, we derived a discrete model, namely, the SGAP-score, through the dichotomization of continuous predictors and the subsequent rounding of Cox-regression β-coefficients. This score has the major strength of being simply applicable in routine practice, without the need for any supporting calculation device. Nevertheless, this obviously came at a cost since the categorization of continuous variables also leads to some disadvantages, such as information loss and the overestimation of differences between subjects close to, but on the opposite sides of, the adopted cut-offs. Keeping these limitations in mind, the application of the SGAP-score still demonstrated good performance for the identification of both low-risk patients and high-risk patients in our cohort. In fact, in our population, patients with an SGAP-score of 0–2 showed a virtually absent risk of recurrence. If further validated on an external cohort, this result may suggest that these patients may be eligible for a less intensive follow-up, e.g., less than annually after 5 years with possible discontinuation after 10 years; this latter suggestion, however, has to be considered with caution, as patients’ follow-up data after this timepoint are limited. On the other hand, patients with an SGAP-score of 5–8 showed a markedly elevated risk of recurrence that exceeded 60% after 10 years and that did not seem to decline over time. This finding may suggest the appropriateness, for these patients, of a more intensive follow-up; in fact, given the observed recurrence rate of ~6% per year within this risk class, a long-term follow-up with biochemical and/or imaging tests every 6 months rather than annually may anticipate the recurrence detection by 6 months in approximately 3 out of every 100 patients each year. Thus, given the significant risks associated with pheochromocytoma recurrence and the potential benefits deriving from its early recognition and treatment, this approach may be of value in improving the long-term post-surgical management of high-risk pheochromocytoma patients.

Our study has some limitations. Clearly, it inherits all limitations already reported for the original model, which were discussed in the reference article [27]; in particular, the available data were insufficient to create different predictive models to distinguish between the risk of local relapse, a new primary tumor, and distant metastases. Nevertheless, although we are aware that these represent different biological entities, the monitoring methods required for the follow-up are essentially the same, and, in clinical practice, the key factor for establishing follow-up periodicity and duration is represented by the recurrence likelihood rather than the recurrence type. Apart from this, the simplification of the predictive model to a predictive score came at some cost: the dichotomization of continuous variables statistically represents a suboptimal choice for model development [31,32,33]. Nevertheless, categorization may often be necessary to allow for the routine use of diagnostic and prognostic tools in clinical practice [31], as excessive complexity unavoidably represents a barrier to their practical application. In the present study, the choice to dichotomize continuous variables was made with the aim of simplifying the clinical application of our predictive tool in routine patient care. Our intention was to obtain a balance between model performance and the simplicity of its use; in this sense, the dichotomization of model variables represents a simplification compared to directly considering the original continuous variables but makes the model much simpler for clinical use. Finally, it must be remarked that an external validation on a different patient cohort is still required for both predictive tools; this point should be addressed in future works so that a definite assessment of their clinical utility can be made.

In conclusion, our study proposed a multivariable scoring system for the prediction of post-surgical recurrence of pheochromocytoma based on the integration of genetic, histopathological, and clinical data, that was derived from a recently published continuous model through a supervised regression and machine-learning approach. A three-class clustering was finally proposed for clinical application and may be of value for comprehensive risk stratification and tailoring post-surgical follow-up in radically operated pheochromocytoma patients.

## Figures and Tables

**Table 1:** SGAP-score point assignment according to multivariable regression coefficients. Abbreviations: PASS, Pheochromocytoma of the Adrenal gland Scaled Score; SGAP, Size–Genetic–Age–PASS.

Parameter	Multivariable Cox Regression Coefficient	Normalized Coefficient	Points for SGAP-Score
Tumor size > 50 mm	+0.4874	$\frac{+0.4874}{+0.4874}=1.0000$	+1
Positive genetic testing	+1.6617	$\frac{+1.6617}{+0.4874}=3.4093$	+3
Age ≤ 35 years	+0.5838	$\frac{+0.5838}{+0.4874}=1.1978$	+1
PASS ≥ 3	+1.4945	$\frac{+1.4945}{+0.4874}=3.0663$	+3

**Table 2:** Predictive performance of the four dichotomous predictive factors and overall SGAP-score. Abbreviations: N/A, not applicable; PASS, Pheochromocytoma of the Adrenal gland Scaled Score; SGAP, Size–Genetic–Age–PASS.

Parameter	Predictive Power Using Somers’ D	*p*-Value for Differences in Predictive Power Compared with the Overall SGAP-Score
Tumor size > 50 mm	0.146	<0.001
Positive genetic testing	0.419	0.033
Age ≤ 35 years	0.294	0.004
PASS ≥ 3	0.220	<0.001
Overall SGAP-score	0.577	N/A

**Table 3:** Recurrence-free survival at 2 years, 5 years, and 10 years according to the SGAP-score risk classes. Abbreviations: CART, classification and regression tree; N, number; SGAP, Size–Genetic–Age–PASS.

Risk Class as Identified Using the CART Algorithm	SGAP-Score	N of Patients	Recurrence-Free Survival at 2 Years	Recurrence-Free Survival at 5 Years	Recurrence-Free Survival at 10 Years
Low risk	0–2	49	100%	100%	100%
Intermediate risk	3–4	97	96%	89%	84%
High risk	5–8	31	74%	70%	37%

## Data Availability

The data presented in this study are available on request from the corresponding author. The data are not publicly available to preserve patient confidentiality.
